# The Effect of Metal Film Thickness on Ignition of Organic Explosives with a Laser Pulse

**DOI:** 10.3390/molecules24244600

**Published:** 2019-12-16

**Authors:** Alexander V. Khaneft, Vadim A. Dolgachev, Svyatoslav A. Rybin

**Affiliations:** 1 Institute of Fundamental Sciences, Kemerovo State University, Kemerovo 650000, Russia; vadimdolgachev@gmail.com (V.A.D.); svyatoslav.a.rybin@gmail.com (S.A.R.); 2 Institute of Power Engineering, National Research Tomsk Polytechnic University, Tomsk 634050, Russia

**Keywords:** simulation, laser ignition, explosive, Al film, Mo film, PETN, RDX, HMX, TATB

## Abstract

The results of numerical ignition simulation of pentaerythritol tetranitrate (PETN), cyclotrimethylene trinitramine (RDX), cyclotetramethylene tetranitramine (HMX) and 1,3,5-triamino-2,4,6-trinitrobenzene (TATB) by aluminium (Al) and molybdenum (Mo) films heated by nanosecond laser pulses in a three-layer system: glass–metal–explosive material (EM) are presented. Influence of metal film thickness on the time of EM ignition delay was considered. A non-linier dependence of time of delay of ignition of EM from thickness of a metal film is shown. The greatest critical thicknesses of Al and Mo metallic films at which ignition of EM is still possible were determined. It was established that the greater the thickness of the metal film and heat resistance of EM, the greater the heat reserve needed in EM ignition film. It was established that the ignition delay time of EM increases in the sequence of PETN, RDX, HMX and TATB.

## 1. Introduction

Experimental and theoretical studies on the numerical simulation of the ignition of explosive compositions have been associated primarily with the creation of lights detonators, whose noise immunity is much higher than that of electric detonators. This is because the laser initiation is immune to electrical signals, which allows one to avoid accidental initiation of explosive material (EM). The second reason for studying the processes of condensed EM ignition is that it is caused by directed regulation of the ignition delay time, and the threshold density of the energy of initiation EM by laser impulses of various duration, intensity, and diameter of the light beam and spectral range [1,2,3,4,5,6,7,8,9,10,11,12,13,14,15,16,17,18]. Organic EM in the impurity absorption region has an insignificant absorption coefficient. Thus, for example, they are practically transparent at the length of a neodymium λ~1.0 μm laser [1]. The initiation threshold of an explosive by a laser pulse is reduced by introducing light-absorbing particles into samples or by applying laser radiation-absorbing films on the explosive surface [2,4,10,14,15,18,19].

The purpose of this paper is to determine the effect of the thickness of the aluminium and molybdenum films that absorb laser pulses on the ignition delay of organic explosives (PETN, RDX, HMX, TATB) in a three-layer system: glass–metal–EM.

## 2. Formulation of the Problem and the Method of Solution

Consider a three-layer heterosystem: glass–metal film–EM. We write down one-dimensional equations of thermal conductivity for this three-layer system. For glass $(h_{1}\geq z\geq 0$):

(1) $$\rho_{1}c_{1}\frac{\partial T_{1}}{\partial t}=\lambda_{1}\frac{\partial^{2}T_{1}}{\partial z^{2}},$$

for metal film $(h_{2}\geq z\geq h_{1}$):

(2) $$\rho_{2}\left[c_{2}+H_{f2}\delta \left(T_{2}-T_{f2}\right)\right]\frac{\partial T_{2}}{\partial t}=\lambda_{2}\frac{\partial^{2}T_{2}}{\partial z^{2}},$$

for EM $(h_{3}\geq z\geq h_{2}$):

(3) $$\rho_{3}\left[c_{3}+H_{f3}\delta \left(T_{3}-T_{f3}\right)\right]\frac{\partial T_{3}}{\partial t}=\lambda_{3}\frac{\partial^{2}T_{3}}{\partial z^{2}}+\rho_{3}QZ\;exp\left(-\frac{E}{RT_{3}}\right),$$

with the initial and boundary conditions:

(4) $$T_{1}\left(z,0\right)=T_{2}\left(z,0\right)=T_{3}\left(z,0\right)=T_{0},$$

(5) $$\frac{\partial T_{1}\left(0,t\right)}{\partial z}=\frac{\partial T_{3}\left(h_{1}+h_{2}+h_{3},t\right)}{\partial z}=0,$$

(6) $$-\lambda_{1}\frac{\partial T_{1}\left(h_{1},t\right)}{\partial z}=\left(1-R_{2}\right)I\left(t\right)-\lambda_{2}\frac{\partial T_{2}\left(h_{1},t\right)}{\partial z},\;T_{1}\left(h_{1},t\right)=T_{2}\left(h_{1},t\right),$$

(7) $$\lambda_{2}\frac{\partial T_{2}\left(h_{1}+h_{2},t\right)}{\partial z}=\lambda_{3}\frac{\partial T_{3}\left(h_{1}+h_{2},t\right)}{\partial z},$$

(8) $$T_{2}\left(h_{1}+h_{2},t\right)=T_{3}\left(h_{1}+h_{2},t\right).$$

where $h_{1}$, $T_{1}$—the thickness and the temperature of the glass plate; $h_{2}$, $T_{2}$—the thickness and the temperature of the metal film, $h_{3}$, $T_{3}$—the thickness and the temperature of EM; $T_{0}$—the initial temperature of the three-layer system glass–metal film–EM; $\lambda_{1}$, $c_{1}$—the thermal conductivity coefficient and the specific heat capacity of the glass plate; $\lambda_{2}$, $c_{2}$—the thermal conductivity coefficient and the specific heat capacity of the metal film; $\lambda_{3}$, $c_{3}$—the thermal conductivity coefficient and the specific heat capacity of the EM; $\rho_{1}$, $\rho_{2}$, $\rho_{3}$ – the densities of the glass, metal film, and EM respectively; $R_{2}$—the reflection coefficients of the light flux from the glass–metal film boundary; $H_{f2}$, $T_{f2}$, $H_{f3}$, $T_{f3}$—heat of fusion and the melting point of the metal film and EM; Q, Z, E—the heat of reaction per unit mass, the frequency factor, and the activation energy of thermal decomposition of EM; R—the universal gas constant; and $\delta (T_{2}-T_{f2})$, $\delta (T_{3}-T_{f3})$—the delta functions.

In the equations of thermal conductivity (Equations (2) and (3)), the functions of the delta are given because, at the temperature of the phase transition $T=T_{f}$ of energy as a function of temperature, they experience jumps in the value of the latent melting points $H_{f2}$ and $H_{f3}$, accordingly. The delta function is given by

$$\delta (T_{i}-T_{fi})=\frac{d\vartheta (T_{i}-T_{fi})}{dT_{i}}$$

where $\vartheta (T_{i}-T_{fi})$ – the Heaviside function defined by the expression

$$\vartheta (T_{i}-T_{fi})=\left\{ \begin{array}{l} 1,\;T_{i}-T_{fi}\geq 0\\ 0,\;T_{i}-T_{fi}<0 \end{array}\right.$$

The algorithm of taking the melting into account in solving the heat conductivity equation for EM is described in [20].

The density of the laser pulse energy flux was set by the expression

$$I(t)=\frac{W}{6\tau_{m}}{\left(\frac{4t}{\tau_{m}}\right)}^{4}\exp\left(-\frac{4t}{\tau_{m}}\right)$$

where W—the energy density of the laser pulse; $\tau_{m}$—the duration of the leading edge of the pulse associated with the duration of the pulse $\tau_{i}=1.19\tau_{m}$ measured as full width at half maximum, and the integral

$$\int_{0}^{\infty }I(t)dt=W$$

The expression for the energy flux density has a maximum at

(9) $$t_{\max}=4\tau_{m}=4.76\tau_{i}$$

This expression is derived from the condition

$${\frac{dI(t)}{dt}|}_{t=t_{\max}}=0$$

Maximum energy flux density

$$I(t_{\max})=\frac{W}{6\tau_{m}}4^{4}\exp(-4)=0.781\frac{W}{\tau_{m}}$$

Absorption of the passed light flux in the glass plate and EM was neglected due to low absorption coefficients of glass and organic EM in this region of the spectrum.

The external heat sink was neglected in this formulation of the EM ignition problem. This is because the duration of the laser pulse and the time of ignition delay is much less than the characteristic time of the external heat sink. In addition, it was assumed that the thermal conductivity and heat capacity of the resulting EM melt and its solid phase are not significantly different. Burnup was also not taken into account, because according to [21], for EM with a high thermal effect, high activation energy and short thermal initiation pulse in the ignition threshold area, the burnup can be ignored. In [22,23], one-dimensional problems of EM ignition by nanosecond pulses of electrons in which burnup was considered were solved. Calculations have shown that EM burnup is insignificant and does not affect the threshold of thermal initiation. For example, for the exponential distribution of the absorbed energy of a nanosecond pulse of electrons, it is shown that at the end of the pulse the degree of decomposition on the surface is ~$3\times 10^{-4}$%, and by the time of explosion ~2.5% [23].

The numerical solution of thermal conductivity equations for glass and Al and Mo metal films was carried out with thermophysical parameters and densities given in Table 1. The numerical solution of the thermal conductivity equation for secondary EM was carried out using kinetic and thermophysical parameters given in Table 2.

At the numerical solution of the equation system (1)–(3) with initial and boundary conditions (4)–(8), the implicit difference scheme was used, which was solved by the sweep method. Arrhenius nonlinearity was linearized at each time step using the Frank–Kamenetsky transformation [24].

Steps on the coordinate $h_{z}$ and time $h_{\tau }$ of the difference scheme were constant regardless of the thickness of the glass plate, metal film and EM. As the preliminary results of numerical calculations have shown, ignition of, for example, PETN occurs near the boundary of the metal film–EM, in the region with a width of about 10 nm. In order to make the temperature distribution curves in the region of EM ignition smooth on the figures, the step on the coordinate $h_{z}=1.0$ nm was chosen. For example, a 100 nm thick metal film was broken into 100 grid points for which difference equations were solved. In the experiment, the thickness of the glass plate and EM is about 1 mm [15].

To reduce the time and calculation error at numerical modeling, the values of glass thicknesses and EM were selected in such a way that by the time of initiation the temperature at the boundaries of the three-layer system stayed unchanged, i.e., $T_{1}(0,t)=T_{3}(h_{1}+h_{2}+h_{3},t)=T_{0}$.

The process of ignition development of EM with 500 nm thick Al film, heated by a nanosecond laser pulse to critical temperature, was, for example, about 0.6 ns for PETN. In order to observe this process, numerical calculations were carried out with the time step $h_{\tau }=0.01$ ns.

When debugging the program in the FORTRAN language, calculations were performed for other values of $h_{\tau }$ as well. For example, at $h_{\tau }=0.02$ ns, at the boundary of the Al film (thickness 100 nm) and PETN at the moment of time t=17.0 ns and temperature of ΔT=646.7 K. At the calculation with step $h_{\tau }=0.01$ ns at t=17.0 ns and temperature of ΔT=645.6 K. The difference between two values is insignificant and makes ~0.19%.

## 3. Results of Numerical Calculations and Their Discussion

Calculations were carried out the duration of laser pulse of $\tau_{i}=30$ ns, energy density $W=3\times 10^{4}$ $J/m^{2}$, light reflection coefficient from the boundary of the glass–metal film of $R_{2}=0.93$ and $T_{0}=300$ K.

**Figure 1:**
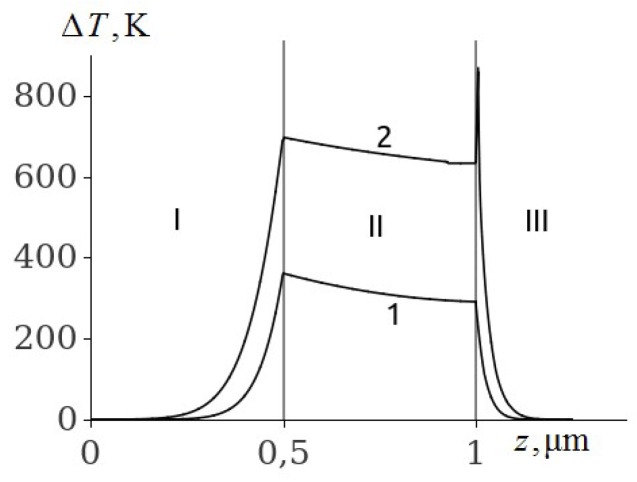
The temperature distribution profile in the glass plate (I), Al film (II), and PETN (III) at the film thickness of $h_{2}=0.5$ μm and time t=20 ns (1), and t=28.8 ns (2).

**Figure 2:**
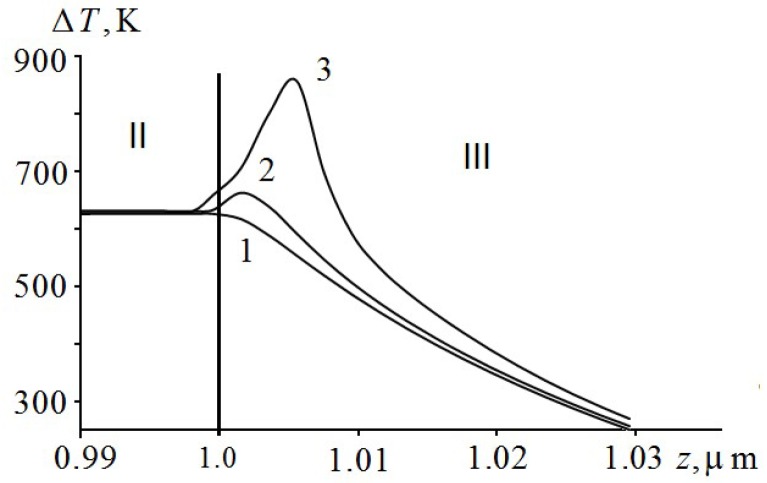
Dynamic distribution of temperature at the boundary Al film (II) and PETN (III) at the film thickness of $h_{2}=0.5$ μm and time t=28.2 ns (1), t=28.4 ns (2) and t=28.8 ns (3).

**Figure 3:**
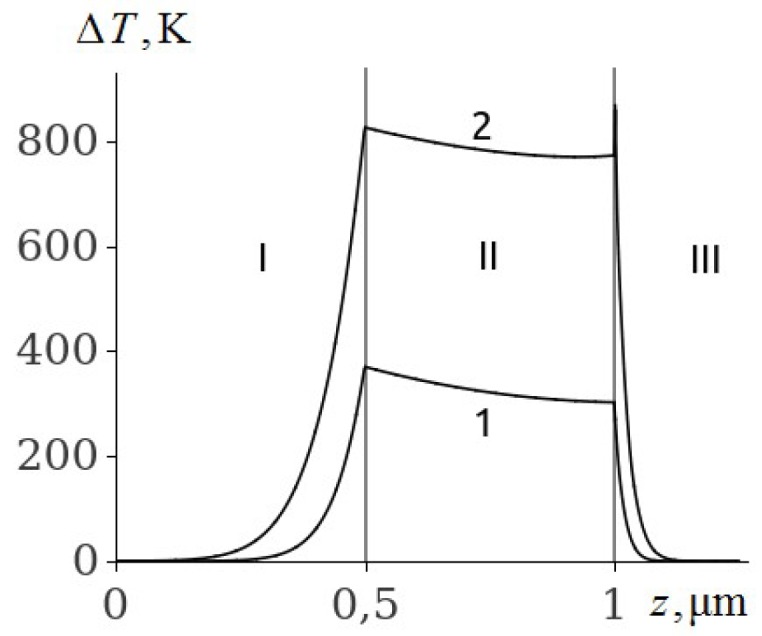
The temperature distribution profile in the glass plate (I), Al film (II), and RDX (III) at the film thickness of $h_{2}=0.5$ μm and time t=20 ns (1), and t=31.35 ns (2).

**Figure 4:**
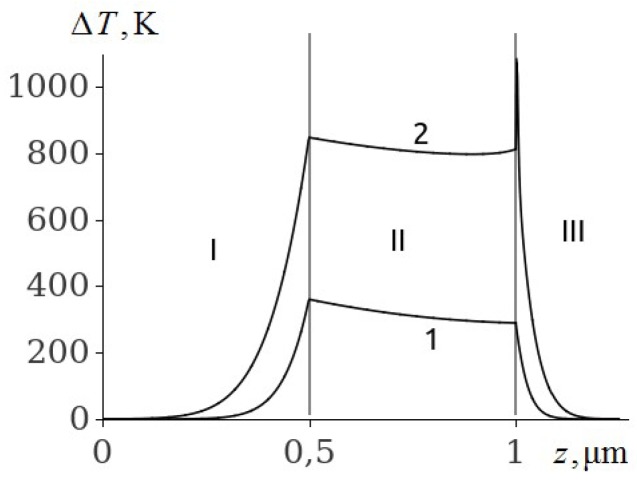
The temperature distribution profile in the glass plate (I), Al film (II), and HMX (III) at the film thickness of $h_{2}=0.5$ μm and time t=20 ns (1), and t=33.315 ns (2).

**Figure 5:**
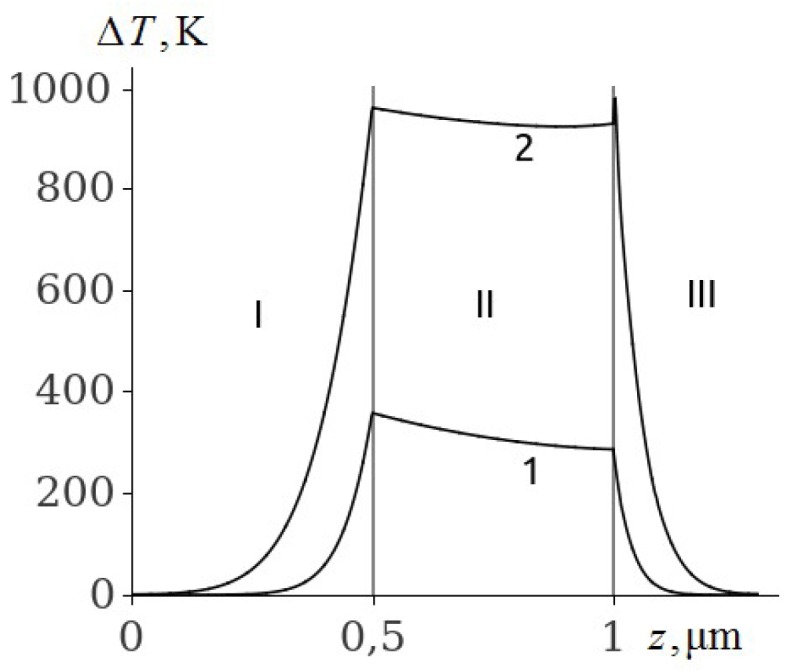
The temperature distribution profile in the glass plate (I), Al film (II), and TATB (III) at the film thickness of $h_{2}=0.5$ μm and time t=20 ns (1), and t=38.05 ns (2).

**Figure 6:**
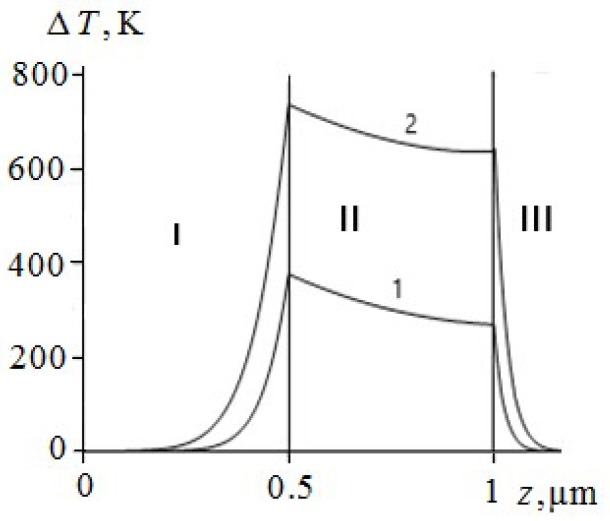
The temperature distribution profile in the glass plate (I), Mo film (II), and PETN (III) at the film thickness of $h_{2}=0.5$ μm and time t=20 ns (1), and t=29 ns (2).

**Figure 7:**
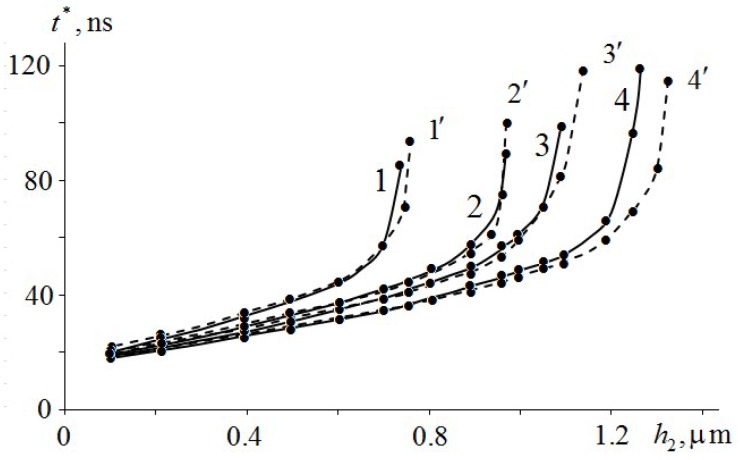
Dependence of the EM ignition delay time on Mo and Al film thickness: TATB (1, 1′), HMX (2, 2′), RDX (3, 3′) and PETN (4, 4′).

Some of the results of the numerical solution of the system of equations of thermal conductivity (1)–(3) with initial and boundary conditions (4)–(8) for PETN, RDX, HMX and TATB are given in Figure 1, Figure 2, Figure 3, Figure 4, Figure 5, Figure 6 and Figure 7. Calculations have shown that the ignition of PETN, RDX, HMX and TATB occurs near the boundary of the metal film–EM (Figure 1, Figure 2, Figure 3, Figure 4, Figure 5 and Figure 6). The film thickness was $h_{2}=0.5$ μm.

It is evident from Figure 1 that there is a plateau on the temperature distribution curve in the aluminum film (curve 2) by the time of the explosion of PETN, caused by the melting of aluminum. On the border with PETN, the aluminium is in a solid state, and on the border with glass it is melted. The PETN ignition delay time according to Figure 1 (curve 2) is 28.8 ns.

Figure 2 shows the dynamics of temperature distribution in the region of EM ignition on a larger scale. As can be seen from Figure 2, the ignition of PETN occurs in a very narrow region of about 10 nm in a short period of time t~0.6 ns. At first, there is a process of self-heating of a near-surface layer by an exothermic reaction (curve 2), passing after 0.4 ns, in ignition of EM. The maximum temperature is shifted by about 9 nm from the interface. The layer in which EM ignition takes place is called the reaction layer [32]. If we considered thermal decomposition of EM, it would have its maximum in this layer [19].

At t>28.8 ns, the system of difference equations of thermal conductivity for EM becomes unstable due to the fact that the adiabatic period of induction $t_{ad}$ in the nodal points of the difference scheme near the interface of the Al film–EM becomes less than the characteristic time of heat diffusion $t_{th}=h_{z}^{2}/a_{3}\sim 10^{-11}$ s from the difference cell. Here, $a_{3}$ are the thermal diffusivity of EM. As can be seen from Table 2, the parameter $a_{3}\sim 10^{-7}$ $m^{2}/s$ for EM and time $t_{th}\approx h_{\tau }$. This leads to the fact that the difference equations of thermal conductivity for EM in the nodal points near the interface are transformed into ordinary differential equations

$$\frac{\partial T_{3i}}{\partial t}=\frac{QZ}{c_{3}}exp\left(-\frac{E}{RT_{3i}}\right)$$

which are unstable. Due to the instability in the system there are pulsations and a chaotic distribution of temperature in the coordinate. Therefore, when analyzing the calculated data, one should be careful not to miss the beginning of instability of the numerical solution of the problem.

The temperature of the aluminium film at ignition of RDX, HMX and TATB (Figure 3, Figure 4 and Figure 5, curve 2) is higher than the melting point. Consequently, the ignition of RDX, HMX and TATB comes from the melting of aluminium. The ignition of RDX and HMX takes place in the range of 5 nm for the time interval t~4.0 ns. The ignition of TATB occurs in the range of about 10 nm for the period of time t~7.0 ns. The ignition delay time of RDX, HMX and TATB according to Figure 3, Figure 4 and Figure 5 (curve 2) is approximately 31.35, 33.315 and 38.05 ns respectively. Thus, the time of ignition delay of EM increases in the sequence of PETN, RDX, HMX and TATB.

From the comparison of the ignition delay times of the EM data under the influence of a nanosecond laser pulse with energy density W=3.0 $J/m^{2}$, the smallest time of ignition delay has PETN, and the largest TATB. The ignition delay time is counted from the beginning of the laser pulse absorption by the three-layer system. 

According to expression (9), at $\tau_{i}=30$ ns:

$$t_{\max}=4\tau_{m}=4.76\tau_{i}=142.8\;\mathrm{ns}$$

Therefore, EM ignition occurs at the front of the laser pulse.

Figure 6 shows the results of the calculation of the temperature distribution in the glass–Mo film–PETN system. Comparison of curves (1) of the temperature distribution in Mo film (Figure 6) and Al film (Figure 1) at t=20 ns shows that the temperature at the glass–Mo film boundary is higher than the temperature at the glass–Al film boundary by 20 K. This is caused by the fact that the temperature conductivity of Al is greater than that of Mo by approximately l.5 times (see Table 1). This effect leads to an increase in the ignition delay time of PETN by 0.2 ns because the fact that the type of metal (Al or Mo) practically does not affect the time of ignition delay of EM due to their high thermal conductivity. Similar calculations were made for RDX, HMX and TATB.

Figure 1 and Figure 3, Figure 4, Figure 5 and Figure 6 show that the temperature distribution curves (1) and (2) in EM are similar. Therefore, it is possible to assume that the heat flux from the metal film into EM $q_{s}\approx \mathrm{const}$. In [32], the criterion of ignition of EM by heat flux density $q_{s}=\mathrm{const}$ and duration $t_{r}$ is obtained assuming that the rate of heat release in a reaction layer with width $z_{r}$ equals the rate of heat removal from a reaction layer at $z=z_{r}$:

(10) $$z_{r}\rho QZexp\left(-\frac{E}{RT_{s}}\right)=-\lambda \frac{dT_{r}}{dz}$$

where $T_{s}$ and $T_{r}$ are the temperature on the surface of EM and on the boundary of the reaction layer, respectively. Thermal decomposition of EM was neglected in [32]. The width of the reaction layer is determined by the condition that the reaction rate on the EM surface is by a factor of *e* higher than on the boundary of the reaction layer [32]. Thus, assuming that $RT_{s}/E<<1$, we can write the following [19]:

(11) $$T_{r}=\frac{T_{s}}{1+RT_{s}/E}\approx T_{s}\left(1-\frac{RT_{s}}{E}\right).$$

The temperature distribution in [32] is determined from the solution of the thermal conductivity equation for inert matter. Taking this solution into account, Equation (10) is written as

(12) $$z_{r}\rho QZ\;exp\left(-\frac{E}{RT_{s}}\right)=q_{s}erfc(y_{0})$$

where $\mathrm{erfc}(y_{0})$ are the additional integral of errors and

(13) $$y_{0}=\frac{z_{r}}{2\sqrt{a_{3}t_{r}}}$$

An expression for determining the thickness of the reaction layer, given that $y_{0}<<1$ has the following form [32]

(14) $$y_{0}=\frac{1-\gamma }{\sqrt{\pi }}$$

where

(15) $$\gamma =\frac{\Delta T_{r}}{\Delta T_{s}}$$

(16) $$\Delta T_{s}=T_{s}-T_{0}=\frac{2q_{s}}{\lambda }\sqrt{\frac{a_{3}t_{r}}{\pi }}$$

Considering Equation (11), Equation (15) can be expressed as

(17) $$\gamma =\frac{\Delta T_{r}}{\Delta T_{s}}=1-\frac{RT_{s}^{2}}{E\Delta T_{s}}$$

Substitute Equation (17) into Equation (14) and, considering Equation (16) and Equation (13), we get the expression for the width of the reaction layer [18]

(18) $$z_{r}=\frac{\lambda }{q_{s}}\frac{RT_{s}^{2}}{E}$$

Furthermore, in (12), we considered that at $y_{0}<<1$ an additional integral of errors $\mathrm{erfc}(y_{0})\sim 1$. As a result, we get the criterion of ignition of EM by constant heat flow

(19) $$\lambda \frac{RT_{s}^{2}}{E}\rho QZ\exp\left(-\frac{E}{RT_{s}}\right)\approx q_{s}^{2}$$

Equation (19) with the accuracy of multiplier “2” agrees with the criterion of ignition of condensed EM by constant heat flow in [33]. This can be explained by the fact that the width of the reaction layer (Equation (18)) is half the width of the reaction layer given in [33].

Equation (19) allows one to calculate the ignition temperature of condensed EM from the known heat flow. Conversely, the known ignition temperature $T_{s}$ is used to estimate the value of the external heat flux $q_{s}$. Equation (19) can be used provided that

$$z_{r}<<h_{3}$$

Another expression for the width of the reaction layer can be obtained from Equations (18) and (19) [19]:

(20) $$z_{r}=\sqrt{a_{3}t_{ad}}$$

where $t_{ad}$ is the adiabatic induction period.

According to Equation (20), the width of the reaction layer is determined by the thermophysical ($a_{3}=\lambda_{3}/c_{3}\rho_{3}$) and kinetic parameters of EM (Z, E). Typical heating time of the reaction layer

$$t_{w}\sim \frac{z_{r}^{2}}{a_{3}}=t_{ad}$$

Figure 7 shows the results of numerical calculations of the dependence of the ignition delay time of TATB (1, 1′), HMX (2, 2′), RDX (3, 3′) and PETN (4, 4′) on the thickness of the Mo and Al films, respectively. As can be seen from Figure 7, the dependence of the ignition delay time $t^{*}$ on the thickness $h_{2}$ of the Mo and Al metal films is non-linear. The asymptote of the curves (1, 1′) – (4, 4′) of the ignition delay time EM from the film thickness is $t^{*}(h_{2}^{*})\to \infty$, where $h_{2}^{*}$ is the critical value of the thickness of the metal film. At $h_{2}\geq h_{2}^{*}$, ignition of EM does not occur because of an insufficient amount of absorbed energy of the laser pulse in a metal film. In addition, one can see in Figure 7 that the type of metal practically does not affect the ignition delay time of EM in the initial section due to its high thermal conductivity. At film thickness $h_{2}\to h_{2}^{*}$, the Mo and Al metal films have a slight effect on the dependence of the ignition delay time of EM on the metal film thickness.

Table 3 shows the $t^{*}$ values for TATB, HMX, RDX and PETN at $h_{2}\approx h_{2}^{*}$ (near the critical thickness), obtained by numerical calculation of EM ignition. We can see that for TATB, HMX, RDX and PETN, $t^{*}$ and $h_{2}^{*}$ are practically independent of the metal type because the thermal diffusivity of metals is three orders of magnitude greater than that of TATB, HMX, RDX and PETN (Table 1 and Table 2). Calculations have shown that the thinner the metal film, the higher its temperature because the energy of the laser pulse is absorbed by the surface. Average temperature of metal film

$$<\Delta T>\sim h_{2}^{-1}$$

Table 3 shows the given values of temperature $\Delta T_{s}$ on the border metal–EM at which it is still possible to neglect the self-heating of EM. These values of $\Delta T_{s}$ are taken from the results of numerical calculations until the moment of ignition, when the EM is heated as an inert body at $h_{2}=h_{2}^{*}$. Only then can Equation (19) be used to estimate the heat flux density at the metal–EM boundary. In addition, Table 3 shows the values of heat flow $q_{s}$ calculated at the temperature $\Delta T_{s}$ using Equation (19). The results of the calculation show (see Figure 7 and Table 3) that TATB has the lowest critical thickness $h_{2}^{*}$ of the metal film. Therefore, it is the most heat-resistant EM. It is followed by HMX, then RDX and PETN. The given dependence of EM temperature stability completely corresponds with the dependence of the critical temperature of thermal explosion of standard TATB, HMX, RDX and PETN [26].

The heat flow $q_{s}$ at which RDX is ignited is smaller than that for PETN, although the temperature $\Delta T_{s}$ is higher. This is due to the thermal diffusivity of RDX being half that of PETN. For more detailed investigation of the ignition conditions of EM in the glass–metal–EM system, additional numerical calculations are required.

## 4. Conclusions

A numerical simulation of ignition of PETN, RDX, HMX and TATB by aluminum and molybdenum films, heated by a nanosecond laser pulse in a three-layer system: glass–metal–EM, was presented. The largest critical thicknesses of Al and Mo films, at which EM ignition is still possible, were determined.

It was established that the greater the thickness of the metal film and the thermal stability of EM, the more heat reserve in the film is needed for ignition of EM. It was shown that the type of metal (Al or Mo) practically does not affect the ignition delay time of EM due to its high thermal diffusivity.

We showed that the type of metal practically does not affect the ignition delay time of EM at $h_{2}<h_{2}^{*}$ due to its high thermal conductivity. At film thickness $h_{2}\to h_{2}^{*}$, the Mo and Al metal have a different effect on the dependence of the ignition delay time of EM on the metal films thickness.

We found that the ignition delay time of EM increases in sequence PETN, RDX, HMX and TATB. By changing the thickness of the metal film, it is possible to directionally control the delay time for the initiation of explosives.

For the critical temperature at the metal–EM interface, determined numerically at the critical thickness of the metal film, the heat flux density was estimated. We found that the lower the thermal diffusivity of EM, the lower the heat flux density is needed for its ignition, as the lower the thermal diffusivity of the substance, the lower the heat flux from the reaction region of EM.

## Figures and Tables

**Table 1:** Thermophysical parameters of materials.

Thermophysical Parameters	Glass	Al	Mo
ρ, $\mathrm{kg}/m^{3}$	2.6 [25]	2.71 [25]	10.21 [25]
c, J/(kg⋅K)	800.0 [25]	896.0 [25]	244.0 [25]
λ, W/(m⋅K)	1.05 [25]	209.0 [25]	135.0 [25]
a, $m^{2}/s$	0.341	8.6	0.54
$H_{f}$, kJ/kg		320.0 [25]	290 [25]
$T_{f}$, K		933.0 [25]	2796.0 [25]

**Table 2:** Kinetic and thermal parameters of organic explosives.

EM	PETN	RDX	HMX	TATB
E, kJ/mol	196.6 [26]	197.3 [26]	220.8 [27]	250.9 [26]
Z, $10^{19}$$s^{-1}$	6.3 [26]	0.202 [26]	5.0 [26]	3.18 [26]
Q, MJ/kg	1.26 [26]	2.1 [26]	2.1 [26]	2.51 [26]
c, $10^{3}$J/(kg⋅K)	1.255 [28]	1.02 [27]	1.25 [10]	1.0 [27]
λ, W/(m⋅K)	0.2508 [26]	0.105 [26]	0.293 [26]	0.418 [26]
ρ, $10^{3}$$\mathrm{kg}/m^{3}$	1.77 [27]	1.82 [29]	1.9 [30]	1.93 [27]
a, $10^{-7}$$m^{2}/s$	1.13	0.57	1.23	2.17
$H_{f}$, kJ/kg	193 [29]	235.5 [29]	192.46 [30]	270 [16]
$T_{f}$, K	413 [26]	476 [26]	558 [26]	> 623 [31]

**Table 3:** Dependence of EM ignition delay time $t^{*}$, temperature $\Delta T_{s}$ and heat flux density $q_{s}$ on the critical metal film thickness $h_{2}^{*}$.

EM		TATB	HMX	RDX	PETN
Al	$h_{2}^{*}$, μm	0.76	0.985	1.125	1.325
	$t^{*}$, ns	94.1	97.0	105.0	112.1
	$\Delta T_{s}$, K	800.0	643.0	626.0	522.0
	$q_{s},10^{9}$ $W/m^{2}$	1.77	1.06	0.624	0.747
Mo	$h_{2}^{*}$, μm	0.75	0.985	1.1	1.275
	$t^{*}$, ns	87.6	92.1	101.9	119.7
	$\Delta T_{s}$, K	801.0	650.0	621.0	532.0
	$q_{s},10^{9}$ $W/m^{2}$	1.8	1.18	0.58	0.896
